# Impact of virtual reality anatomy training on ultrasound competency development: A randomized controlled trial

**DOI:** 10.1371/journal.pone.0242731

**Published:** 2020-11-23

**Authors:** Kai-Chun Hu, Daniel Salcedo, Yi-No Kang, Che-Wei Lin, Chin-Wang Hsu, Chung-Yi Cheng, Fat-Moon Suk, Wen-Cheng Huang

**Affiliations:** 1 Department of Emergency, School of Medicine, College of Medicine, Taipei Medical University, Taipei, Taiwan; 2 Department of Emergency and Critical Medicine, Wan Fang Hospital, Taipei Medical University, Taipei, Taiwan; 3 Department of Clinical Education, Wan Fang Hospital, Taipei Medical University, Taipei, Taiwan; 4 Center for Education in Medical Simulation, Taipei Medical University, Taipei, Taiwan; 5 Department of Education and Humanities in Medicine, School of Medicine, Taipei Medical University, Taipei, Taiwan; 6 Department of Internal Medicine, Division of Nephrology, Wan Fang Hospital, Taipei Medical University, Taipei, Taiwan; 7 Department of Internal Medicine, School of Medicine, College of Medicine, Taipei Medical University, Taipei, Taiwan; 8 Department of Internal Medicine, Division of Gastroenterology, Wan Fang Hospital, Taipei Medical University, Taipei, Taiwan; Waseda University, JAPAN

## Abstract

**Purpose:**

The use of Virtual Reality (VR) in health professions education has increased dramatically in recent years, yet there is limited evidence of its impact on educational outcomes. The purpose of the study was to assess the impact of VR anatomy instruction on the ultrasound competency of novice learners participating in a ultrasonography workshop.

**Method:**

We designed a VR-enhanced ultrasonography training program and utilized a plane transection tool to interact with a three-dimensional (3D) VR model of the human body which facilitated the 3D conceptualization of the spatial relationship of anatomical structures, leading to faster and better development of ultrasonographic competency. This was a randomized control study which enrolled third-year medical students (n = 101) without previous exposure to formal or informal ultrasonography training. The participants were randomly divided into an intervention and control group. We assessed participants’ competency through ultrasound performance stations on live subjects, we also measured anatomical and ultrasound image identification ability using multiple choice tests.

**Result:**

Participants in the intervention group (median = 16; interquartile 13 to 19) had significantly higher scores in ultrasonography task performance tests than the control group (median = 10; interquartile 7 to 14; Mann-Whitney U = 595; P < 0.01). In sub-group analysis, the intervention group performed significantly better in the six out of ten ultrasound tasks. Participants in the intervention group also had greater improvement in ultrasonographic image identification MCQ tests than the control group (Mann-Whitney U = 914; P < 0.05).

**Conclusion:**

This study suggests that VR-enhanced anatomical training could be of significant benefit in ultrasonography training by promoting a better understanding of the spatial relationships of anatomical structures and the development of early psychomotor skills transferable to the handling of ultrasonographic probes.

## Introduction

Point-of-Care Ultrasonography (POCUS) is an essential tool in medical practice due to its ability to enhance the procedural and diagnostic skills of a clinical practitioner [1]. The use of POCUS in health professions education can support the learning of important clinical concepts in preclinical education, such as the development of a better understanding of the spatial relationship of anatomical structures [2]. Early exposure to ultrasonographic training has been shown to have several benefits, including the development of a more practical, clinically oriented understanding of anatomy and physiology, early development of psychomotor skills to facilitate the learning of procedural tasks, and highlighting the importance of POCUS in clinical practice [3]. A systematic review of 65 studies revealed that learners, in general, have a positive perception of the use of ultrasound in undergraduate medical education. Students’ also demonstrated a willingness to include ultrasonography as part of their early training [4].

In addition to deliberate practice for mastering ultrasound skills, learners require the mental integration of multiple bidimensional (2D) images to construct a three-dimensional (3D) perception of anatomy and pathology [5]. The variability of the development of these mental constructs may result in individual variation in the acquisition of procedural competence. For most novices, the acquisition of competence in ultrasonography is challenging due to the knowledge transfer from 2D to 3D anatomical perception of the human body [6]. The immersive and interactive nature of virtual reality (VR), promotes a clearer and deeper understanding of the 3D relationships between anatomical structures [7]. VR can support learners in developing a realistic and effective mental construct of the spatial relationship of anatomical structures before or after ultrasound training [8]. The literature suggests that VR can help learners develop more accurate anatomical localization skills, as well as reduce the overall cognitive load of ultrasonography training by establishing clear connections between previously acquired anatomy knowledge and images observed by ultrasonography [9,10].

Accordingly, highly interactive VR can increase learners’ motivation and its applicability to education [11–13]. The immersion and interactivity of VR can allow learners to gain a more complete understanding of the spatial relationships of objects (such as anatomical structures) in the virtual environment [14]. In addition, when VR is applied to learning, the higher level of immersion in the virtual environment, allows a greater learning effect to be achieved [15]. VR can also simulate visual, auditory, and other sensory modalities allowing it to mimic the real world, thereby facilitating the conceptualization of a functional mental model [16].

The access to low-cost and high-resolution VR devices now allows learning to occur through hands-on immersive virtual experiences in many professional fields, including military, aviation and healthcare [17]. A meta-analysis conducted by Kyaw et al. concluded that VR has the potential to transform health professions education by improving both knowledge and skills, and highlighted the need for additional research to evaluate the outcomes of VR in terms of learner attitudes and satisfaction, as well as the cost-effectiveness of this technology and its potential to modify clinical practice behaviours [18]. Many studies had proved that VR is helpful for preoperative preparation for surgical teams as well as anatomy learning for medical students [19,20], but few studies have looked into the specific benefits of VR in the development of ultrasonography skills.

In 1956 Bloom et al. [21] developed a taxonomy of educational objectives which included 3 major domains: Cognitive, Affective and Psychomotor. The Psychomotor domain was not developed in detail until 1975 when Dave et al. [22] published behavioral objectives for the psychomotor domain. Additional versions of this taxonomy were developed by other authors [23,24] (S1 Table). We used original version as the theoretical framework for the design of both the VR and non-VR components of the ultrasonography training program developed for this study.

The purpose of the study was to assess the impact of VR-enhanced learning on the ultrasound competency of novice learners without previous exposure to ultrasound training. We hypothesized that the use of a plane transection tool in a three-dimensional VR model of the human body would aid in the transfer of psychomotor skills necessary for the adequate manipulation of the ultrasonographic probes and contribute to the conceptualization of the spatial relationship of anatomical structures in the thorax and abdomen.

We were also interested in determining the effect of VR anatomy training on anatomy and ultrasonographic image recall questions.

## Method

We utilized a randomized control study design to evaluate the impact of VR-assisted anatomical training on ultrasonographic competency development in an ultrasound educational intervention in learners with no previous experience in this field.

### Study population

This study enrolled third-year medical students (Taiwan has a 6-year undergraduate medical program) enrolled at Taipei Medical University School of Medicine, who had successfully completed all human anatomy courses.

Our exclusion criteria were previous formal or informal ultrasonography training.

**Fig 1:**
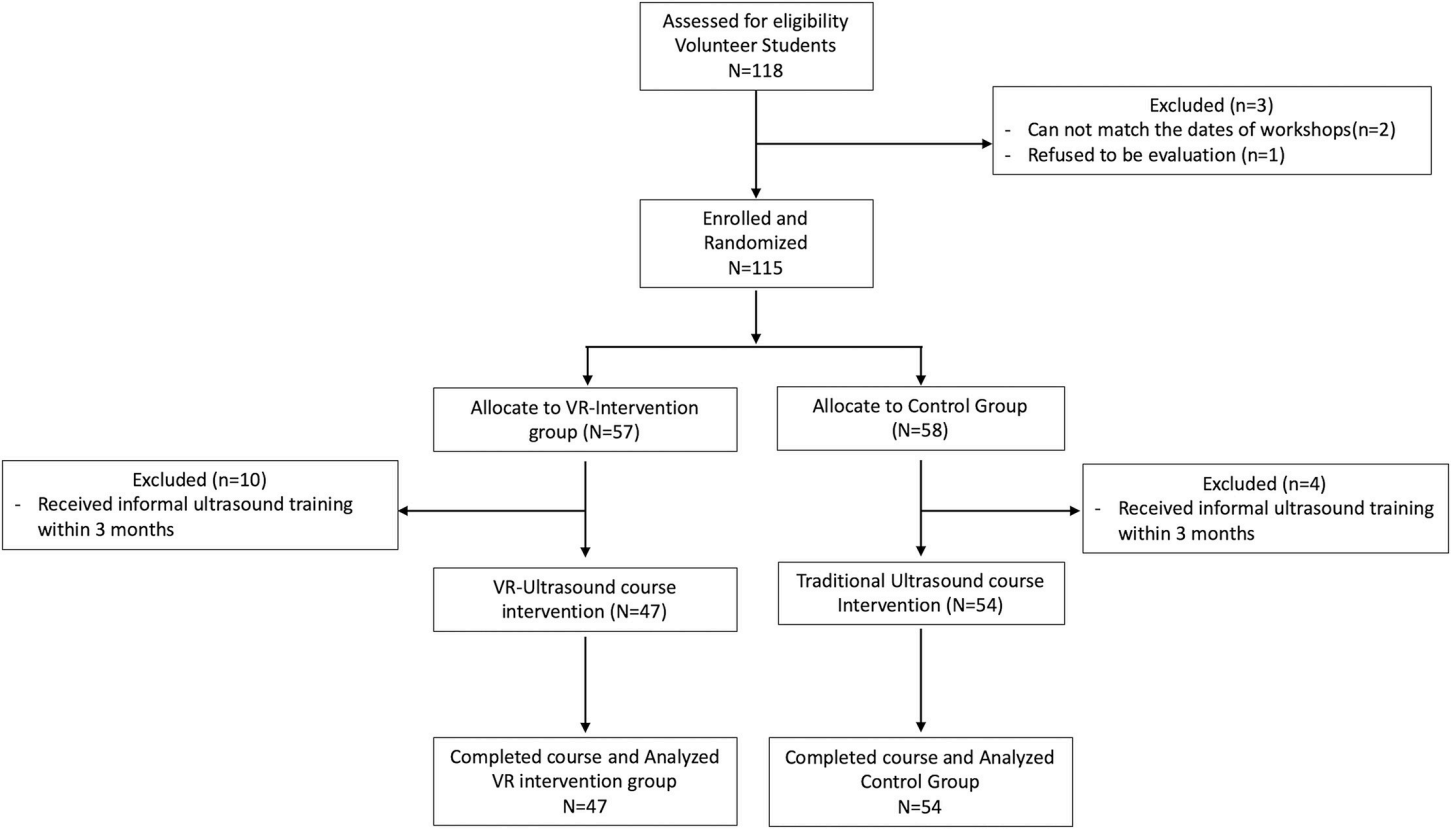
Randomization flow chart.

A total of 115 participants (61 male and 54 female) were recruited by email announcement, and allocated into two groups, a VR intervention group (n = 57) and a control group (n = 58). 14 participants dropped out (10 from the intervention group and 4 from the control group) due to participation in informal ultrasound training after initial recruitment. A total of 101 participants participated in this study. (Fig 1) Participants in the VR intervention group used VR as part of their training during the course, including a self-directed VR-enhanced anatomy review of thorax and abdomen, and additional VR review sessions during ultrasonography hands-on practice. The participants in the control group took part in an ultrasound workshop of similar design. The VR anatomy component was replaced with a review session using a digital atlas. Upon conclusion of the workshop all participants (control and intervention groups) were assessed using a standardized practical multi-station ultrasonography test.

The workshops were conducted between October 2019 and December 2019.

The research protocol was approved by The Joint Institutional Review Board of Taipei Medical University (Approval No.: TMU-JIRB N201911040), and participants signed a consent form agreeing to participate in the study.

### Curriculum design

**Fig 2:**
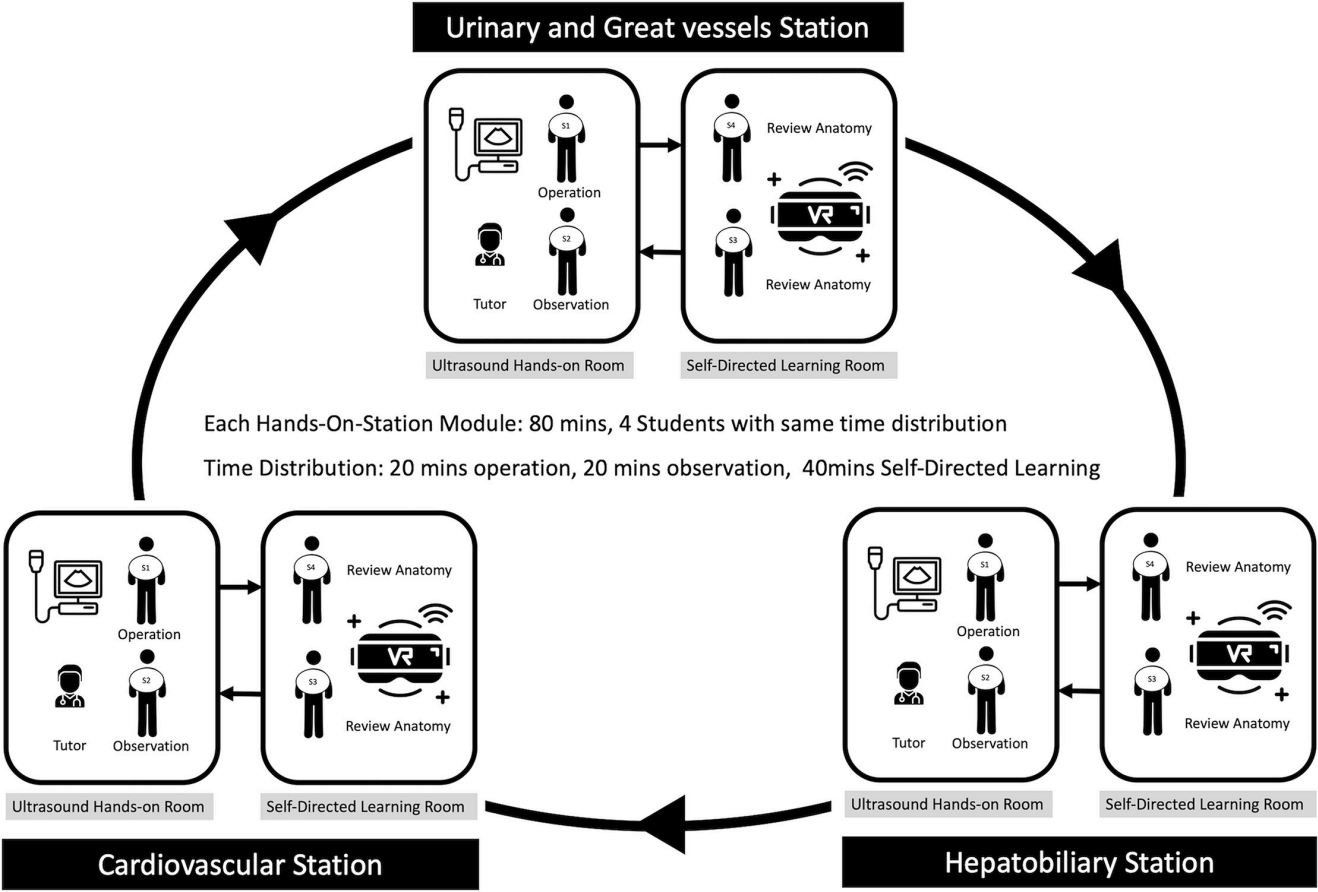
Time and sequence of the VR intervention group.

Both intervention and control courses were designed as a 6-hour practical workshop (Fig 2). The workshop design was based on Peyton’s 4-step approach method optimized for ultrasonographic skill training [25]. An initial instructional lecture was provided to introduce the basic concepts and operation of ultrasonographic equipment. Participants in both control and intervention groups were provided a checklist of anatomical structures including the urinary, vascular, hepatobiliary, and cardiovascular systems, and instructed to capture screenshots of each structure (S2 Table). The control group captured screenshots of an electronic atlas of human anatomy [26], while the VR intervention group took screenshots of an interactive 3D model of the human body. Each group was allotted 60 minutes to complete the assignment and screenshots were evaluated by the instructors. The practical ultrasonography training portion of the workshop consisted of three stations focusing on the systems included in the anatomical checklist. In clinical practice, the cardiovascular and hepatobiliary ultrasonographic exams are considered more complex and technically challenging.

### Materials and equipment

The VR anatomy software used in this study was the Anatomy Master module of MedicalHolodeck (MedicalHolodeck, Switzerland), which provides VR anatomical models. This VR software offers the ability to transect anatomical models in any angle using a VR controller. This function is similar to the manipulation of an ultrasound probe. The software also allows users to easily take screenshots and save them for later review.

The VR hardware used in this study was the VIVE Pro headset (1440x1600 resolution, 90Hz refresh rate) with dual controllers (HTC Vive, Taiwan).

The ultrasonography equipment used at each station was the Sonosite SII (FUJIFILM Sonosite Inc.), including abdominal probes (rC60Xi, 2–5 MHz Curved) and cardiac probes (rP19x,1–5 MHz Phased).

### Outcome measurements and data collection instruments

This study included the development and use of 2 assessment instruments and 2 checklists. We developed a 10 multiple choice question (MCQ) 2D anatomic image test to assess the ability to identify relevant structures and a 10 question MCQ ultrasound anatomy test focusing on the identification of anatomical structures in static ultrasonographic images. The questions on both MCQ tests were based on the anatomical structure checklist (S2 Table) provided to participants.

The anatomical structure checklist items included the structures evaluated in the three ultrasonographic instructional modules (cardiovascular, hepatobiliary, urinary and great vessels of abdomen).

The ultrasonographic instructional modules and performance assessment checklists (S3 Table) were developed following the recommendations of POCUS Ultrasound-American College of Emergency Physicians (ACEP) Policy Statement 2016 [27].

In addition, operational scoring (Score 0- unable to position the probe and/or performing basic ultrasound tasks, score 1-can partially complete basic ultrasound identification. The station items and objectives were developed based on the operations and obtained images, and score 2—can correctly perform ultrasound tasks and obtain accurate images) was utilized to measure performance in the assessment tool.

This study included pre and post-testing of both anatomy and ultrasound images. The station items and objectives were developed based on the consensus of 11 experts in POCUS in line with the ACEP Policy Statement.

### Statistical analysis

Before the analysis, we determined the change in score of written tests in ultrasonography and anatomy by calculating differences between post-test and pre-test, and explored data distribution using Kolmogorov-Smirnova test. Since our data on performance time, task competency, and written test scores did not have a normal distribution, descriptive statistics portion of our study focused on the median, interquartile, and interquartile range (IQR).

We used crosstab to examine the gender differences between the intervention group and control group, and conducted a Mann-Whitney U test to examine the differences in performance time, task competency, and written test scores of anatomy and ultrasonography between the two groups. Since the data distribution was not normal (S4 Table). Cohen’s *d* was estimated based on Mann-Whitney U statistics and sample size before power testing. G*Power was used for power analysis. The Mann-Whitney U test in G*Power is a subgroup within the t-test family, and we chose “Post hoc” for testing power by using two tails estimates with the given effect size (Cohen’s *d*), alpha error probability, and sample size. Threshold for statistical significance in this study was based on a common cut-point 0.05 for *P* value.

## Result

### Demographics

101 medical students were randomized into intervention (n = 47) and control (n = 54) groups. The intervention group involved 25 males and 22 females, the control group involved 27 males and 27 females (Table 1). The two groups had a similar age and gender ratio (Chi-square = 0.171; P > 0.05).

**Table 1:** Demographic of 101 participants.

Baseline	Control group (n = 54)	Intervention group (n = 47)
Age	21	21
Gender		
Male; n (%)	27 (50.0)	25 (53.2)
Female; n (%)	27 (50.0)	22 (46.8)
Unspecified; n (%)	0 (0)	0 (0)
MCQ test before course		
Ultrasonographic images; median (range)	4 (1 to 8)	4 (1 to 7)
Anatomic images; median (range)	8 (5 to 10)	7 (3 to 9)

Participants in the intervention group had slightly lower score in pre-test of anatomic images than those in the control group (mean difference = -1.19; P < 0.05), while their score in pre-test of ultrasonographic images was similar to score in the control group (mean difference = -0.17; P > 0.05).

### Primary outcomes

The maximum time allowed for ultrasonographic tasks was 600 seconds in both groups. Although students in the intervention group spent slightly shorter time than those in the control group, we did not find significant differences in time spent between the two groups (Table 2). Mann-Whitney U test demonstrated that students in the intervention group had significantly higher scores of ultrasonographic task performance than in the control group (Mann-Whitney U = 595; *P* < 0.01). In further analysis, median scores of ultrasonographic task performance in the intervention group and control group were 16 and 10 respectively, nine out of ten task items in ultrasonographic task performance were scores as “Completely Done” in the intervention group, but in the control group, only four out of ten task items were scored as “Completely Done” (Table 3).

**Table 2:** Difference in primary outcomes and secondary outcomes between intervention group and control group.

	Control group	Intervention group	M-W		Cohen’s	Observed
Outcome	Mean rank	Mean rank	*U*	*P*	*d*	power
**Primary outcomes**						
Time spent	54.23	47.29	1094.5	0.12	--	--
US performance (Overall)	38.52	65.34	595	< 0.01	1.03	1
Inferior vena cava	47.81	54.67	1096.5	0.12	--	--
Abdominal aorta	39.33	64.40	639	< 0.01	0.94	1
Morrison pouch	48.30	54.11	1123	0.20	--	--
Douglas pouch	44.56	58.39	921.5	< 0.01	0.48	0.64
Pericardial space (Subxiphoid view)	43.22	59.94	849	< 0.01	0.59	0.82
Spleen	46.93	55.68	1049	0.09	--	--
Kidney	46.67	55.98	1035	0.07	--	--
Gallbladder (Subcostal view)	42.17	61.15	792	< 0.01	0.68	0.91
Main portal vein	43.81	59.26	881	< 0.01	0.55	0.76
Heart (Parasternal long axis view)	43.11	60.06	843	< 0.01	0.60	0.83
Secondary outcomes						
Ultrasonographic images (Post-test)	44.43	58.55	914	0.01	0.50	0.68
Ultrasonographic images (Change)	45.28	57.57	960	0.03	0.43	0.55
Anatomic images (Post-test)	56.26	44.96	985	0.04	0.39	0.47
Anatomic images (Change)	43.07	60.11	841	< 0.01	0.61	0.84

M-W, Mann-Whitney test; Cohen’s d and observed power was only performed for findings with statistical significance.

**Table 3:** Descriptive statistics of ultrasonographic task performance and the change of written tests score in each group.

	Control group			Intervention group		
Outcome	Median	Quartile 1	Quartile 3	Median	Quartile 1	Quartile 3
**Primary outcome**						
Time spent	600	600	600	600	570	600
US performance (Overall)	10	7	14	16	13	19
Inferior vena cava	2	1	2	2	2	2
Abdominal aorta	1	0	2	2	2	2
Morrison pouch	2	1	2	2	2	2
Douglas pouch	2	1	2	2	2	2
Pericardial space (Subxiphoid view)	1	0	2	2	1	2
Spleen	1	0	2	2	1	2
Kidney	2	0	2	2	1	2
Gallbladder (Subcostal view)	1	0	2	2	1	2
Main portal vein	0	0	2	2	0	2
Heart (Parasternal long axis view)	0	0	1	1	0	2
**Secondary outcome**						
Ultrasonographic images (Change)	2	0	3	3	1	4
Anatomical images (Change)	0	0	1	1	0	2

Significant differences between the intervention and control groups could be observed in six out of ten ultrasonographic task items, and scores of these six items were higher in the intervention group (Table 2). However, the observed power for two of the six items was < 0.8. The intervention group performed significantly better in six ultrasonographic visualization tasks including abdominal aorta (Mann-Whitney U = 639; *P* < 0.01), douglas pouch (Mann-Whitney U = 921.5; *P* < 0.01), pericardial space (Mann-Whitney U = 849; *P* < 0.01), gallbladder (Mann-Whitney U = 792; *P* < 0.01), main portal vein (Mann-Whitney U = 881; *P* < 0.01), and heart under parasternal long axis view (Mann-Whitney U = 843; *P* < 0.01). Effect sizes (Cohen’s *d*) for the significant findings ranged between 0.48 and 0.94, and observed powers were between 0.64 and 1.

### Secondary outcome

Students in the intervention group (median = 3, IQR = 3) demonstrated greater improvement in test scores of ultrasonographic images than the control group (median = 2, IQR = 3; Mann-Whitney U = 960; P < 0.05) (Tables 2 and 3). Students in the intervention group also obtained higher scores in the written test of ultrasonography image identification after the course (Mann-Whitney U = 914; *P* < 0.05). These two significant findings appear to be underpowered.

Participants in the intervention group showed greater improvement in non-ultrasound anatomical image tests (median = 0, IQR = 1; Mann-Whitney U = 841; *P* < 0.05) with observed power > 0.8. Although they still had significantly lower scores in non-ultrasound anatomic image tests (Mann-Whitney U = 985; *P* < 0.05) post-intervention.

## Discussion

This study evaluated the impact of applying VR to support the training of ultrasonography skills. The transfer of theoretical anatomical knowledge to imaging has always been challenging in ultrasonography, where the appropriate visualization of anatomical structures is highly dependent on the operator’s skill [28,29]. The results obtained in this study indicate the use of VR anatomy training has a positive impact on the learning of ultrasonography skills in learners with no previous experience performing bedside ultrasound. The integration of VR in the educational intervention resulted in improved performance in test station checklist results, which was particularly evident in stations considered to be of higher difficulty such as cardiac and hepatobiliary ultrasonography.

### Improved performance in ultrasonographic testing stations

Test data obtained from participants after completing the course indicate superior overall performance of the VR intervention group in identifying target anatomical structures in healthy individuals. A sub-analysis of these results suggests that this difference is more significant in stations considered to be more technically challenging, such as the visualization of the cardiovascular system and hepatobiliary tract. This increased complexity is related to the specific location, size and movement of target structures. Individual station checklist results indicate that learners in the VR intervention group performed better in challenging stations than their non-VR counterparts. These findings are in line with our hypothesis that VR anatomy training not only provides learners with a better theoretical understanding of regional anatomy but also promotes the development of psychomotor skills that aid in the performance of ultrasonographic tasks. The VR anatomical pre-training allows learners to use the dominant-hand controller to manipulate the digital model using hand motions similar to those observed while handling an ultrasonographic probe. By applying the principles of the psychomotor domain of Bloom’s taxonomy (Simpson, 1972), we theorize that VR anatomical training improves learner’s ability to use sensory cues to guide motor activity (Perception), increases action readiness (Set) and the ability to learn complex motor skills by imitation, trial and error (Guided response). These findings are also in line with the psychomotor domain taxonomy developed by Dave in 1975, in which VR could aid in the imitation, manipulation, precision and articulation of complex movements, helping learners develop the skills necessary to successfully locate and correctly identify target structures in ultrasonographic examinations.

### Target structure ultrasonographic image acquisition time

The results also indicated that learners in the VR intervention group were able to visualize anatomical structures in healthy humans in less time than their Non-VR counterparts. The time difference was not statistically significant, further analysis suggested that the difference in time was more evident in certain individuals in the VR group, who were consistently able to identify target anatomical structures in a shorter time. It is of importance to highlight that these changes were not consistent within the entire population of the VR intervention group and that the results may be influenced by the general psychomotor ability of individual participants. Another aspect not contemplated in this study was the confidence level of participants to correctly identify target anatomical structures, which could be an interesting parameter for further study.

### Anatomy testing results

Participants from both intervention and control groups completed a pre and post anatomical knowledge test to evaluate the theoretical knowledge of ultrasonographic anatomy. In line with our hypothesis, the VR intervention group demonstrated greater improvement of scores between the pre and post-test, which suggests that the ability to visualize anatomical structures in virtual reality may provide better understanding of 3D relationships of anatomical structures. This may be related to the ability to transect the VR anatomical model in plains similar to those obtained through ultrasonography. These findings suggest that the use of VR for learning focused regional anatomy may help gain a better awareness of the disposition and spatial relationship of anatomical structures, and an enhanced understanding of ultrasonographic visualization windows.

### The role of VR-enhanced anatomical training in ultrasonographic skill development

The increasing availability of low-cost VR hardware and applications for health professions education allows VR to be easily integrated into ultrasonographic training. The immersive and interactive elements of this technology allow the development of both cognitive and psychomotor skills, leading to significantly better results in global ultrasound competency development among novice learners. VR-enhanced anatomy can help overcome the gap from theoretical anatomical knowledge to the clinical application of these concepts, which are necessary for the practice of bedside ultrasonography. These practical concepts include spatial relationships of anatomical structures and a better understanding of visualization plains.

### Study limitations

Regardless of the randomization methods used in this study, the generalizability of these results may be affected by learners’ previous knowledge of regional anatomy and their general psychomotor ability. As demonstrated in the analysis of the time taken to identify specific target structures, there are individual performance differences among participants regardless of the method of training, and identifying specific factors affecting these differences is a potential area of future study.

The sample size of this study was determined to be sufficient to obtain accurate results, but further study with larger samples and a wider variety of learners might confirm some of the findings of this study.

Finally, this study focused on the identification of normal target structures in healthy individuals, but the impact of VR anatomy training on the ability to identify pathological findings has not yet been determined, and could be a field for future study.

In conclusion, this study aimed to identify the impact of using virtual reality to enhance the development of ultrasonography skills among novice learners. The results of this study suggest that VR-enhanced anatomical training could be of significant benefit in ultrasonography training by promoting a better understanding of the spatial relationships of anatomical structures and the development of early psychomotor skills transferable to the handling of ultrasonographic probes.

## Supporting information

S1 Table Psychomotor domain of Bloom’s taxonomy model. (PDF)

S2 Table Anatomical structure checklist. (PDF)

S3 Table Performance assessment checklist. (PDF)

S4 Table Descriptive statistics and normal distribution test. (PDF)
