# Supplementary material for: Impact of virtual reality anatomy training on ultrasound competency development: A randomized controlled trial
Source: PLoS One. 2020 Nov 23;15(11):e0242731. doi: 10.1371/journal.pone.0242731 (PMC7682883; doi:10.1371/journal.pone.0242731)
Supplement: S1 Table — (PDF) [file pone.0242731.s001.pdf]

**S1 Table.** Psychomotor domain of Bloom's taxonomy model

| **Dave, 1975** | | **Simpson, 1972** | | **Harrow, 1972** | |
|---|---|---|---|---|---|
| Category | Key concept | Category | Key concept | Category | Key concept |
| Imitation | Performing a skill while observing a demonstrator | Perception | Ability to use sensory cues to guide motor activity | Reflex movements | involuntary reaction |
| Manipulation | perform a skill on one's own after taking lessons or reading about it | Set | Shows desire to learn a new process | Fundament movements | Perform a simple task |
| Precision | Perform a skill or task without assistance | Guided Response | Imitation and trial and error | Perceptual abilities | Track a moving object |
| Articulation | Combining a series of skills or activities to meet a novel requirement | Mechanism | Skills can be performed with confidence and proficiency | Physical abilities | Strength and agility |
| Naturalization | Mastering a high-level performance | Complex Overt Response | Skillful performance of the skills | Skilled movements | Advanced learned movements |
| | | Adaptation | Skills can modify to fit special requirements | Nondiscursive communication | Use effective body language |

**Reference**


Dave, R.H. (1975). *Developing and writing behavioral objectives*. (R J Armstrong, ed.) Educational Innovators Press.

Simpson E.J. The Classification of Educational Objectives in the Psychomotor Domain. 1972. doi:10.1007/978-1-4419-1428-6_6020

Harrow, A.J. (1972). *A taxonomy of the psychomotor domain*. New York: David McKay Co.
