# Supplementary material for: Impact of virtual reality anatomy training on ultrasound competency development: A randomized controlled trial
Source: PLoS One. 2020 Nov 23;15(11):e0242731. doi: 10.1371/journal.pone.0242731 (PMC7682883; doi:10.1371/journal.pone.0242731)
Supplement: S2 Table — (PDF) [file pone.0242731.s002.pdf]

S2 Table. Anatomical structure checklist

Module 1

1	Hepato-renal fossa (Morison's pouch)
2	Spleno-renal recess
3	Douglas pouch or recto-vesicular pouch (Transverse)
4	Douglas pouch or recto-vesicular pouch (Sagittal)
5	Subxiphoid: pericardial cavity (identify RV/LV)
6	Abdominal aorta scan to bifurcation (Transverse)
7	Abdominal aorta with celiac trunk and superior mesenteric artery (Sagittal)
8	Inferior vena cava: diameter (Sagittal)

Module 2

9	Right kidney: cortex/medulla/renal pelvis (Coronal)
10	Left kidney: cortex/medulla/renal pelvis (Coronal)
11	Bladder: Long/Short axis
12	Gallbladder and wall thickness
13	Portal vein: Left/Right/Main
14	Hepatic vein: Right/Middle/Left
15	Portal triad: portal vein/hepatic artery/biliary tract (Transverse)
16	Spleen with hilum (Coronal)
17	Splenic vein (Transverse)
18	Pancreas

Module 3

19	PLAX: pericardial cavity
20	PLAX: right ventricle and left ventricle eyeballing contractility
21	PLAX: aorta root and measure diameter
22	PSAX: Papillary Muscle Level: papillary muscle
23	PSAX: Mitral Valve Level: mitral valve
24	PSAX: Aortic Valve Level: right atrium/right ventricle/left atrium/aorta
25	Apical four: right atrium/right ventricle/left atrium/left ventricle
