# Supplementary material for: Impact of virtual reality anatomy training on ultrasound competency development: A randomized controlled trial
Source: PLoS One. 2020 Nov 23;15(11):e0242731. doi: 10.1371/journal.pone.0242731 (PMC7682883; doi:10.1371/journal.pone.0242731)
Supplement: S3 Table — (PDF) [file pone.0242731.s003.pdf]

S3 Table. Performance assessment checklist

Ultrasound competency evaluation form				
Organ/Landmark	View	Not done[a] (0 point)	Partial done[b] (1 point)	Completely done[c] (2 point)
1.Inferior Vena Cava	Sagittal view			
2.Abdominal Aorta	Trace Aorta from upper Abdomen to bifurcation			
3.Morrison pouch	Coronal view			
4.Douglas Pouch	Transverse View			
5.Pericardial Space	Subxiphoid view			
6.Spleen (with hilum)	Coronal view			
7.Kidney (with cortex, medulla, renal pelvis)	Left side, coronal view			
8.Gall bladder	Subcostal View			
9.Main portal vein	Subcostal View			
10. Heart (LA, RV, LV, aortic root)	Parasternal long axis View			

[a] Not done coded as scanning the wrong structure.

[b] Partial done coded as one of the following:

1) Scan the correct anatomical structure with poor quality of image or incomplete anatomical structure.
2) Wrong orientation of probe

[c] Complete done coded as scanning the correct as well as fully anatomical structure with correct orientation of probe. Also, image quality is good without shadow.
