# Supplementary material for: Impact of virtual reality anatomy training on ultrasound competency development: A randomized controlled trial
Source: PLoS One. 2020 Nov 23;15(11):e0242731. doi: 10.1371/journal.pone.0242731 (PMC7682883; doi:10.1371/journal.pone.0242731)
Supplement: S4 Table — (PDF) [file pone.0242731.s004.pdf]

S4 Table. Descriptive statistics and normal distribution test

					K-S test	
Outcome	Minimum	Maximum	*M*	*SD*	*Statistic*	*P*-value
Primary outcome						
Time spent	360	600	581.35	44.29	0.41	< 0.01
US performance (Overall)	3	20	12.97	4.81	0.13	< 0.01
Inferior vena cava	0	2	1.56	0.78	0.45	< 0.01
Abdominal aorta	0	2	1.36	0.81	0.35	< 0.01
Morrison pouch	0	2	1.67	0.58	0.44	< 0.01
Douglas pouch	0	2	1.52	0.72	0.40	< 0.01
Pericardial space (Subxiphoid view)	0	2	1.17	0.80	0.27	< 0.01
Spleen	0	2	1.33	0.84	0.35	< 0.01
Kidney	0	2	1.38	0.83	0.38	< 0.01
Gallbladder (Subcostal view)	0	2	1.12	0.93	0.32	< 0.01
Main portal vein	0	2	1.06	0.96	0.32	< 0.01
Heart (Parasternal long axis view)	0	2	0.80	0.85	0.30	< 0.01
Secondary outcome (MCQ test)						
Ultrasonographic images (Change)	-2	5	0.98	1.30	0.21	< 0.01
Anatomic images (Change)	-2	6	2.31	1.90	0.13	< 0.01

K-S, Kolmogorov-Smirnova; M, mean; SD, standard deviation.
